# Supplementary material for: Oral Administration of a Novel, Synthetic Ketogenic Compound Elevates Blood β-Hydroxybutyrate Levels in Mice in Both Fasted and Fed Conditions
Source: Nutrients. 2024 Oct 18;16(20):3526. doi: 10.3390/nu16203526 (PMC11510390; doi:10.3390/nu16203526)
Supplement: Supplementary file 1 [file nutrients-16-03526-s001.zip › nutrients-3158935-supplementary.pdf]

F1: Supplemental Figure S1. Proton NMR spectrum of product 1 shown in Figure 2: propane-1, 2, 3-triyl tris (3-oxobutanoate), common name, glycerol-tri-acetoacetate (Gly-3AcAc). NMR analysis was performed using a Bruker Neo 400 MHz spectrometer.

^{1}H NMR (400 MHz, Chloroform-*d*) δ 5.38 – 5.28 (m, 1H), 4.37 (d, *J* = 4.2 Hz, 1H), 4.34 (d, *J* = 4.2 Hz, 1H), 4.28 (d, *J* = 6.2 Hz, 1H), 4.25 (d, *J* = 6.2 Hz, 1H), 3.43 (s, 6H), 2.17 (s, 9H)

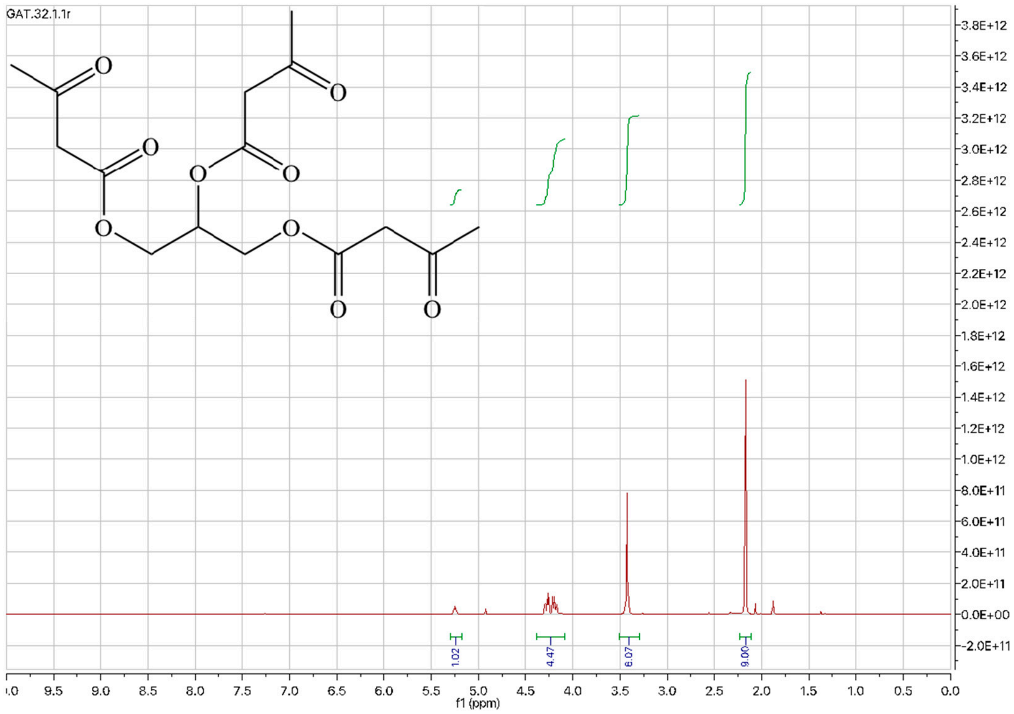

Figure S1.

Table S1: Exp 1 BHB Cmax

	Cmax	Cmax
Animal	Value	Time (hrs)
Mouse 1	0.80	4
Mouse 2	0.90	1
Mouse 3	0.80	2
Mouse 4	0.80	24
Mouse 5	0.70	3
Mouse 6	1.30	1
Mouse 7	1.40	1
Mouse 8	1.40	1
Mouse 9	1.80	1
Mouse 10	1.20	1
Mouse 11	2.20	3
Mouse 12	1.60	1
Mouse 13	1.80	3
Mouse 14	2.00	3
Mouse 15	1.80	5
Mouse 16	2.40	5
Mouse 17	3.20	3
Mouse 18	2.40	4
Mouse 19	2.30	1
Mouse 20	2.60	4

Table S2: Exp 2 BHB Cmax

	Cmax	Cmax
Animal	Value	Time (hrs)
Mouse 1	1.40	20
Mouse 2	1.40	20
Mouse 3	1.40	20
Mouse 4	1.70	16
Mouse 5	1.70	16
Mouse 6	1.60	1
Mouse 7	3.10	4
Mouse 8	2.40	4
Mouse 9	1.60	2
Mouse 10	1.70	12

Table S3: Exp 4 BHB Cmax

	Cmax	Cmax
Animal	Value	Time (hrs)
Mouse 1	0.7	1
Mouse 2	0.8	1
Mouse 3	0.4	2
Mouse 4	0.5	1
Mouse 5	0.7	1
Mouse 6	1.1	4
Mouse 7	3.1	2
Mouse 8	1.3	2
Mouse 9	4	2
Mouse 10	2	2
